# Efficacy and Safety of Intensified Versus Standard Prophylactic Anticoagulation Therapy in Patients With Coronavirus Disease 2019: A Systematic Review and Meta-Analysis

**DOI:** 10.1093/ofid/ofac285

**Published:** 2022-06-07

**Authors:** Nicola K Wills, Nikhil Nair, Kashyap Patel, Omaike Sikder, Marguerite Adriaanse, John Eikelboom, Sean Wasserman

**Affiliations:** Department of Medicine, University of Cape Town, Cape Town, South Africa; Michael G. DeGroote School of Medicine, McMaster University, Hamilton, Ontario, Canada; School of Medicine, University of Ottawa, Ottawa, Ontario, Canada; Michael G. DeGroote School of Medicine, McMaster University, Hamilton, Ontario, Canada; Department of Medicine, University of Cape Town, Cape Town, South Africa; Michael G. DeGroote School of Medicine, McMaster University, Hamilton, Ontario, Canada; Division of Infectious Diseases and HIV Medicine, Department of Medicine, University of Cape Town, Cape Town, South Africa; Wellcome Centre for Infectious Diseases Research in Africa, Institute of Infectious Disease and Molecular Medicine, University of Cape Town, Cape Town, South Africa

**Keywords:** intensified anticoagulation, COVID-19, mortality, thrombosis, bleeding

## Abstract

**Background:**

Randomized controlled trials (RCTs) have reported inconsistent effects from intensified anticoagulation on clinical outcomes in coronavirus disease 2019 (COVID-19). We performed an aggregate data meta-analysis from available trials to quantify effect on nonfatal and fatal outcomes and identify subgroups who may benefit.

**Methods:**

We searched multiple databases for RCTs comparing intensified (intermediate or therapeutic dose) vs prophylactic anticoagulation in adults with laboratory-confirmed COVID-19 through 19 January 2022. We used random-effects meta-analysis to estimate pooled risk ratios for mortality, thrombotic, and bleeding events (at end of follow-up or discharge) and performed subgroup analysis for clinical setting and dose of intensified anticoagulation.

**Results:**

Eleven RCTs were included (N = 5873). Intensified vs prophylactic anticoagulation was not associated with a mortality reduction up to 45 days (risk ratio [RR], 0.93 [95% confidence interval {CI}, .79–1.10]). There was a possible signal of mortality reduction for non–intensive care unit (ICU) patients, although with low precision and high heterogeneity (5 studies; RR, 0.84 [95% CI, .49–1.44]; *I*^2^ = 75%). Risk of venous thromboembolism was reduced (RR, 0.53 [95% CI, .41–.69]; *I*^2^ = 0%), with effect driven by therapeutic rather than intermediate dosing (interaction *P* = .04). Major bleeding was increased with intensified anticoagulation (RR, 1.73 [95% CI, 1.17–2.56]) with no interaction for dosing and clinical setting.

**Conclusions:**

Intensified anticoagulation has no effect on mortality among hospitalized adults with COVID-19 and is associated with increased bleeding risk. The observed reduction in venous thromboembolism risk and trend toward reduced mortality in non-ICU settings requires exploration in additional RCTs.

**Clinical Trials Registration.** CRD42021273449 (PROSPERO).

**Key Points:** In a meta-analysis of 11 trials comparing intensified to prophylactic anticoagulation in 5873 adults with COVID-19, overall, no effect on short-term mortality was shown despite a significant reduction in venous thromboembolic events.

Coronavirus disease 2019 (COVID-19) is associated with increased risk of venous and arterial thrombotic events [1, 2], particularly in patients with severe disease [3], with incidence rates even higher than those seen in historical cohorts of critically ill individuals with non–COVID-19 respiratory disease [4]. Venous thrombotic risk remains high even with use of standard prophylactic anticoagulation [3]. The interplay of direct viral-induced endothelial injury with a dysregulated inflammation response and coagulation factor activation are postulated as key contributors to the development of the COVID-19–associated prothrombotic state [5–7]. Thrombo-inflammation has been linked to disease progression and poor outcomes in patients with COVID-19 [6, 8]; in particular, increased circulating D-dimer (a biomarker of inflammation and coagulation activation) is an independent predictor of mortality [9–11].

These observations led to widespread use of therapeutic anticoagulation in patients hospitalized with COVID-19, especially heparin, which is believed to have anti-inflammatory and antiviral properties [12, 13], in the hope it may prevent thrombotic events and improve outcomes. Some noncomparative studies suggested that intensified (intermediate or therapeutic)–dose anticoagulation may reduce thrombotic complications [14, 15], but cohort studies with matched controls did not show mortality benefit [16, 17] and higher bleeding risk has been consistently reported [18, 19]. Observational studies are limited by the potential for confounding as well as noncomparability across study populations, selection and observer bias, and inconsistent ascertainment of key outcomes, leaving major uncertainty around risk-benefit.

Randomized controlled trials (RCTs) offer more robust estimates of treatment effect. However, most RCTs of anticoagulation strategies for COVID-19 have been small, enrolling several hundred rather than thousands of participants, and were not powered to assess important individual clinical outcomes. Three RCTs, enrolling between 300 and 700 participants per treatment arm, were neutral for primary composite outcomes that included both thrombotic events and mortality and did not demonstrate mortality benefit with intensified anticoagulation, and only 1 of these trials showed a reduction in thrombotic events [20–22]. A larger RCT involving non–critically ill patients (n = 2219) [23] hospitalized with COVID-19 found that intensified therapy compared with usual-dose thromboprophylaxis reduced need for organ support and major thrombosis, but not overall mortality. A small effect with low precision in this single positive trial, inconsistent effects across different studies, and a strong reproducible signal of increased bleeding risk limit definitive conclusions around use of intensified anticoagulation in COVID-19. Synthesizing evidence from all available RCTs may provide more precise estimates of effect and identify subgroups that derive the greatest absolute benefit from intensified anticoagulation. Additional power from pooled data may also enable separate examination of the effects of treatment on individual outcomes, for example, thrombotic events and mortality, potentially providing insights into the prognostic importance of thrombosis. We undertook a systematic review and aggregate data meta-analysis to obtain best estimates of the effect of intensified vs standard prophylactic anticoagulation on clinically important outcomes for patients with COVID-19.

## METHODS

### Eligibility Criteria

We included RCTs comparing intensified, defined as intermediate (generally 1 mg/kg of enoxaparin once daily, or an equivalent) or therapeutic dosing, vs standard prophylactic dose anticoagulation for adults with laboratory-confirmed COVID-19 (Table 1). No restriction by language, publication status (including articles in preprint), anticoagulation agent, or clinical setting was applied (Supplementary Table 1). We only included studies reporting at least 1 of the prespecified outcomes listed in Table 1.

**Table 1.** PICOT Eligibility Criteria for Study Inclusion

PICOT element		Eligibility criteria
Population		Adults with laboratory-confirmed COVID-19, receiving care in any clinical setting (outpatient or inpatient, including non-ICU and ICU-level care)
Intervention and comparator/exposure		Intermediate-dose prophylactic anticoagulation vs standard low-dose prophylaxisTherapeutic anticoagulation vs standard low-dose prophylaxis
Outcomes	Primary	All-cause mortality at 30 days, death, or discharge^a^
	Secondary	At 30 days, death, or discharge, rates of:
		1. Venous thromboembolism
		2. Pulmonary embolism
		3. Deep venous thrombosis
		4. Any arterial thrombosis
		5. Any thrombosis
		6. Composite outcome of thrombosis or death
		7. Days requiring organ support
		8. Any requirement for respiratory support (IMV or ECMO)
		9. Major bleeding
		10. Clinically relevant nonmajor bleeding
		11. Major or clinically relevant nonmajor bleeding
		12. Any bleeding

Abbreviations: COVID-19, coronavirus disease 2019; ECMO, extracorporeal membrane oxygenation; ICU, intensive care unit; IMV, invasive mechanical ventilation; PICOT, population, intervention, comparator, outcomes and timing criteria.

a Study deviations from these predefined timepoints have been described in the analysis.

### Search Strategy

An electronic search was conducted on 19 September 2021 and repeated on 19 January 2022 using Medline (PubMed), Scopus, the World Health Organization (WHO) COVID-19 database (https://search.bvsalud.org/global-literature-on-novel-coronavirus-2019-ncov/), and the Cochrane Library. We also screened the WHO Trial Registry Network (https://trialsearch.who.int/) and ClinicalTrials.gov (https://clinicaltrials.gov/) for ongoing/recently completed trials, and the International Prospective Register of Systematic Reviews (PROSPERO; https://www.crd.york.ac.uk/PROSPERO/) for ongoing or recently completed systematic reviews. We searched preprint literature by scanning the WHO COVID-19 database as well as the National Institutes of Health iSearch COVID-19 portfolio (https://icite.od.nih.gov/covid19/search/). A search strategy was developed using multiple terms relating to anticoagulation, anticoagulant agents, and COVID-19 (Supplementary Table 2).

### Record Management and Data Extraction

Records from the primary search were entered into Mendeley reference management software version 1.19.8 (https://www.mendeley.com/) and duplicates removed. Titles and abstracts were screened against the study eligibility criteria (Table 1) by K. P., N. N., and O. S. and independently by M. A. and N. K. W., followed by review of the full texts of potentially eligible articles for inclusion. After consensus on studies meeting criteria for inclusion, variables of interest (Supplementary Table 3) were extracted on a Microsoft Excel spreadsheet by N. N. and O. S. with independent verification by M. A. and N. K. W. Reference lists of included studies were screened to identify any additional eligible studies. Risk of bias in individual studies was independently assessed by K. P., M. A., and N. K. W. using version 2 of the Cochrane risk of bias tool for randomized trials (https://training.cochrane.org/handbook/current/chapter-08), with respect to the key outcome of interest (mortality). S. W. and J. E. were consulted for review of any conflict regarding study inclusion, data discrepancies, or assessing risk of bias.

### Data Analysis

The primary outcome was all-cause mortality at end of follow-up or discharge. Other efficacy outcomes of interest included venous thromboembolism (symptomatic or asymptomatic VTE, including pulmonary embolism [PE] or deep vein thrombosis [DVT]), arterial thrombosis (stroke, myocardial infarction, acute limb ischemia, other arterial ischemia), any thrombotic event, and a composite of thrombosis or death. The key safety outcome was major bleeding; other safety outcomes included clinically relevant nonmajor bleeding and any bleeding event. We planned to analyze the effect of intensified anticoagulation on days requiring any organ support and respiratory support (invasive mechanical ventilation or extracorporeal membrane oxygenation), but these outcomes were not reported by included trials.

We performed an intention-to-treat analysis (the denominator was all randomized participants who received at least 1 dose of assigned treatment). Data were pooled using a random-effects meta-analysis model with restricted maximum likelihood estimation. We computed risk ratios (RRs) with 95% confidence interval (CI) as measures of effect. Between-study heterogeneity was quantified using the *I*^2^ statistic [24]. Sensitivity analysis using the “leave-one-out” approach was done to visually evaluate the influence of each study on the overall pooled effect for mortality. We performed prespecified subgroup analysis for baseline severity of illness (intensive care unit [ICU] setting vs general ward [where >50% of randomized participants admitted in general ward]) and dose of intensified anticoagulation (therapeutic vs intermediate doses). Funnel plots were generated to assess publication bias for each of the primary and secondary outcomes. All meta-analyses were performed using Stata 17 software.

## RESULTS

### Characteristics of Included Studies

**Figure 1.**
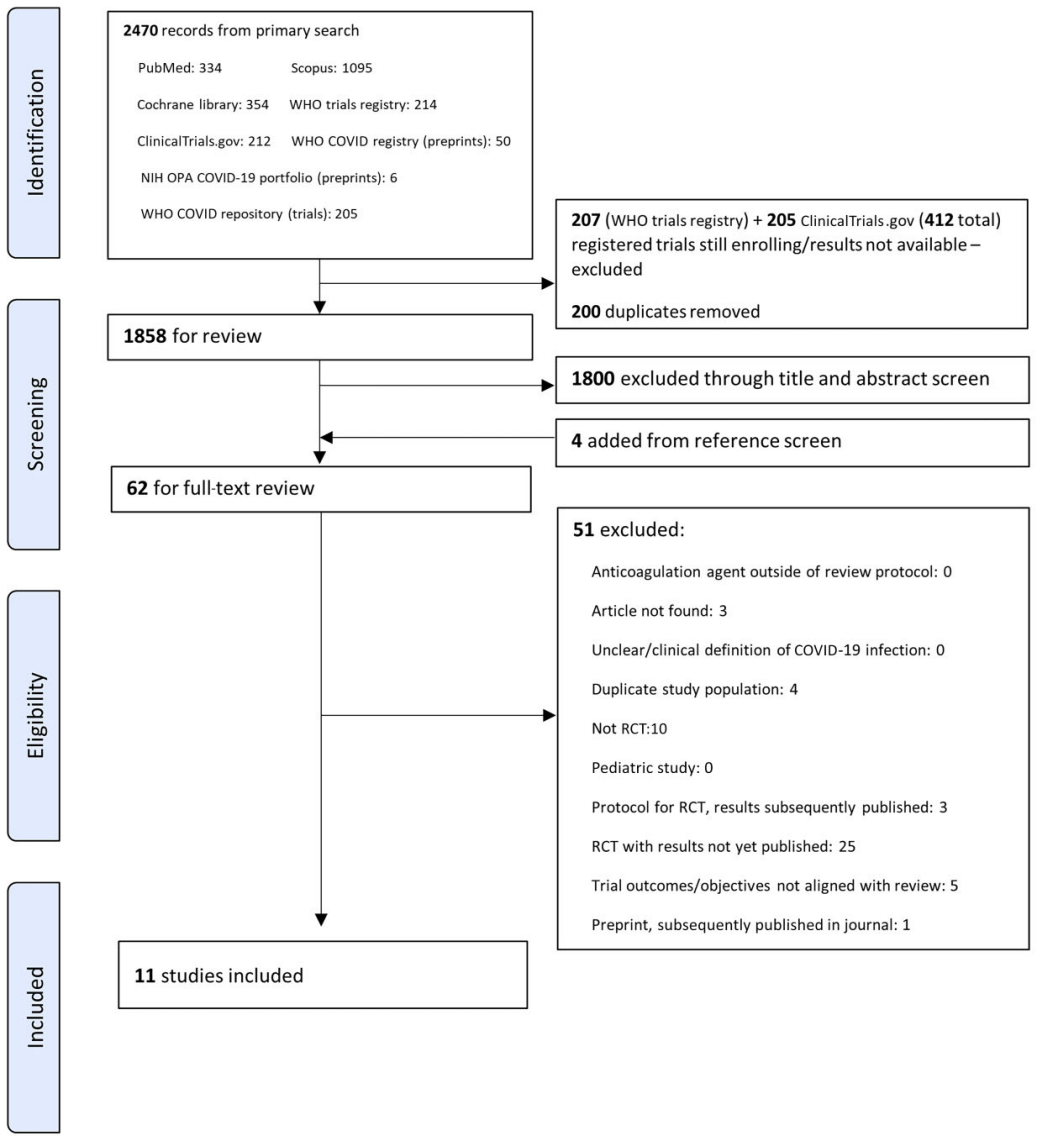
Preferred Reporting Items for Systematic Reviews and Meta-Analyses (PRISMA) diagram. Abbreviations: COVID-19, coronavirus disease 2019; NIH, National Institutes of Health; OPA, Office of Portfolio Analysis; RCT, randomized controlled trial; WHO, World Health Organization.

We screened 2470 records and included 11 studies meeting eligibility criteria (Figure 1); these studies contributed data from 5873 adults with confirmed COVID-19 who were followed up over a median of 30 days (range, 21–45 days). Key information from included studies is summarized in Table 2 with full study details provided in Supplementary Tables 4–6.

**Table 2.** Key Details of Included Studies

Study	Setting	Enrollment Period	Intervention	Comparator	Primary Outcome (Follow-up Period)	Sample Size^a^
INSPIRATION [21]	ICU; Iran	Jul 2020–Nov 2020	Intermediate-dose enoxaparin	Standard low-dose enoxaparin prophylaxis	Composite outcome: symptomatic VTE or ATE, ECMO treatment, or death (30 days)^b^	562
REMAP-CAP, ACTIV-4a, and ATTACC (non–critically ill) [23]	Hospitalized, non-ICU; 9 countries^c^	Apr 2020–Jan 2021	Therapeutic enoxaparin or UFH	Usual care thromboprophylaxis (low-dose or intermediate-dose enoxaparin/UFH)	In-hospital death and organ support–free days (21 days)	2219
REMAP-CAP, ACTIV-4a, and ATTACC (critically ill) [20]	ICU-level support; 9 countries^c^	Apr 2020–Jan 2021	Therapeutic enoxaparin or UFH	Usual care thromboprophylaxis (low-dose or intermediate-dose enoxaparin/UFH)	In-hospital death and organ support–free days (21 days)	1098
RAPID [25]	Hospitalized, non-ICU with elevated D-dimer; 6 countries^d^	May 2020–Apr 2021	Therapeutic LMWH or UFH	Standard low-dose prophylaxis (LMWH or UFH)	Composite: death, mechanical ventilation, ICU admission (28 days)	465
HEP-COVID [26]	Hospitalized, requiring oxygen, with elevated D-dimer or coagulopathy (33% in ICU); USA	May 2020–Apr 2021	Therapeutic enoxaparin	Standard low-dose or intermediate-dose enoxaparin/UFH	ATE, symptomatic or asymptomatic VTE or death (30 ± 2 days)^e^	253
ACTIV-4B [27]	Outpatient; USA	Sep 2020–Jun 2021	Therapeutic apixaban	Prophylactic low-dose apixaban	Composite: Symptomatic VTE or ATE, hospitalization for CVS or pulmonary events, or death (45 days)	278
ACTION [22]	Hospitalized with elevated D-dimer levels (6% in ICU); Brazil	Jun 2020–Feb 2021	Therapeutic rivaroxaban or enoxaparin	Standard low-dose prophylaxis with enoxaparin/LMWH	Composite: time to death, duration of hospitalization, or duration of supplemental oxygen (30 days)	614
Perepu et al [28]	ICU or with laboratory- confirmed coagulopathy; USA	Apr 2020–Jan 2021	Intermediate-dose enoxaparin	Standard low-dose prophylactic enoxaparin	All-cause mortality (30 days)	173
HESACOVID [29]	ICU; Brazil	Apr 2020–Jul 2020	Therapeutic enoxaparin	Standard low-dose prophylactic enoxaparin/UFH	Gas exchange variations (PaO_2_:FiO_2_) (baseline, 7 and 14 days)^f^	20
BEMICOP [30]	Hospitalized, non-ICU, with elevated D-dimer; Spain	Oct 2020–May 2021	Therapeutic bemiparin	Standard bemiparin prophylaxis	Composite: death, ICU admission, mechanical ventilation, moderate/severe ARDS, or symptomatic VTE/ATE (30 days)^g^	65
Oliynyk et al [31]	ICU with elevated D-dimer, nonventilated; Ukraine	Jul 2020–Mar 2021	Therapeutic LMWH or UFH	Standard low-dose enoxaparin prophylaxis	Rates of intubation and death (28 days)	126

Abbreviations: ACTION, AntiCoagulaTloncOroNavirus trial; ACTIV, Accelerating COVID-19 Therapeutic Interventions and Vaccines; ARDS, acute respiratory distress syndrome; ATE, arterial thromboembolism; ATTACC, AntithromboticTherapy to Ameliorate Complications of Covid-19; BEMICOP, Comparison of Two Different Doses of Bemiparin in COVID-19; CVS, cardiovascular system; ECMO, extracorporeal membrane oxygenation; HEP-COVID, Full Dose Heparin Vs. Prophylactic Or Intermediate Dose Heparin in High Risk COVID-19 Patients; ICU, intensive care unit; HESACOVID, Full versus prophylactic heparinization for the treatment of severe forms of SARS-Covid-19; INSPIRATION, Intermediate versus Standard-dose Prophylactic anticoagulation In cRitically-ill pATIents with COVID-19: An opeN label randomized controlled trial; LMWH, low-molecular-weight heparin; PaO_2_:FiO_2_, ratio of partial pressure of oxygen in arterial blood to fractional inspired oxygen; RAPID, Therapeutic Anticoagulation versus Standard Care as a Rapid Response to the COVID-19 Pandemic trial; REMAP-CAP, Randomized, Embedded, Multifactorial Adaptive Platform Trial for Community-Acquired Pneumonia; UFH, unfractionated heparin; USA, United States of America; VTE, venous thromboembolism.

a Intention-to-treat population (denominator all randomized participants who received at least 1 dose of assigned treatment).

b The INSPIRATION trial published independent reports on 30- and 90-day outcomes; for the purposes of this review, only 30-day outcomes were included.

c USA, Canada, United Kingdom, Brazil, Mexico, Nepal, Australia, The Netherlands, Spain.

d Brazil, Canada, Ireland, Saudi Arabia, United Arab Emirates, USA.

e Only trial to specify screening for asymptomatic deep venous thrombosis with Doppler compression ultrasonography at 10 + 4 days or at discharge if sooner and if no symptomatic VTE event prior to this point.

f Secondary outcomes: in-hospital mortality and bleeding at 28 days.

g Ten-day safety outcomes reported and included in meta-analysis.

Five ICU-based studies reported outcomes among 1979 critically ill patients [20, 21, 28, 29, 31], 5 studies reported outcomes from 3616 patients hospitalized in a general ward setting [22, 23, 25, 26, 30], and 1 study reported outcomes from 278 outpatients [27]. Nine studies (n = 5138) [20, 22, 23, 25–27, 29–31] compared therapeutic low-molecular-weight heparin (LMWH), unfractionated heparin, or rivaroxaban/apixaban to standard thromboprophylaxis (3 inpatient studies allowed either standard low-dose or intermediate-dose enoxaparin in the “usual care” comparator arm [20, 23, 26]). In the remaining 2 studies (n = 735) [21, 28], both conducted in an ICU setting, intermediate-dose enoxaparin was compared to standard-dose enoxaparin thromboprophylaxis.

Median age ranged from 52 to 71 years and 41% of patients were female (11 studies, n = 5873) with median body mass index ranging from 26 kg/m^2^ to 34 kg/m^2^ (10 studies, n = 5747). Thirty-eight percent were prescribed an antiviral agent at baseline (8 studies, n = 5004) and 64% received corticosteroids at baseline (9 studies, n = 5469). Hypertension was reported in 45% (9 studies, n = 4659) and diabetes in 30% (10 studies, n = 5747). Chronic lung or cardiovascular disease was documented in 17% and 8% of patients, respectively (9 studies, n = 5469).

Risk of bias assessment is reported in Supplementary Table 7 and Supplementary Figure 1): 4 studies had a low risk of bias, 2 were assessed as high risk, and 5 had some concerns. Funnel plot for the mortality outcome showed some asymmetry, suggesting possible publication bias, but the number of included studies was small (Supplementary Figure 2).

### Primary Outcome

**Figure 2.**
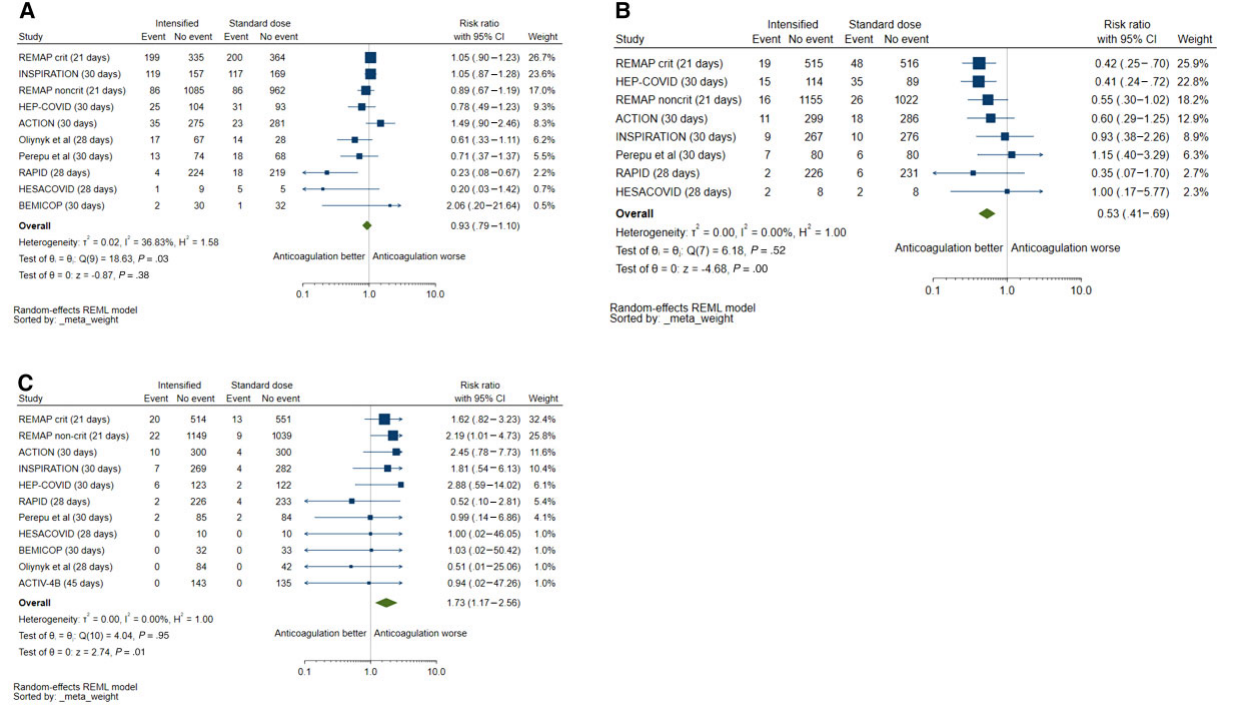
*A*, Mortality with intensified vs prophylactic anticoagulation. The single outpatient trial [27] was excluded from the forest plot because of no mortality events. *B*, Venous thromboembolism with intensified vs prophylactic anticoagulation. The single outpatient trial [27] was excluded from the forest plot because of no mortality events. Two other trials were excluded because venous thromboembolic events were not captured as outcomes [30, 31]. *C*, Major bleeding with intensified vs prophylactic anticoagulation. Abbreviations: CI, confidence interval; REML, restricted maximum likelihood.

Eleven studies were included for the primary outcome of all-cause mortality: 16.7% (501/3004) died in the intensified anticoagulation group and 17.9% (513/2869) died in the prophylactic anticoagulation group. Intensified anticoagulation was not associated with a reduction in mortality for up to 45 days compared with prophylactic anticoagulation (RR, 0.93 [95% CI, .79–1.10]). There was significant heterogeneity, with 37% of variability in effect size estimates due to between-study differences (*P* = .03; Figure 2*A*). On sensitivity analysis, omission of individual trials had no significant influence on pooled mortality (Supplementary Figure 3).

### Secondary Efficacy Outcomes

Only 1 study (n = 253) [26] screened for asymptomatic DVT with Doppler compression ultrasonography, but the majority of reported VTE events were symptomatic. Symptomatology was not specified in the REMAP-CAP (Randomized, Embedded, Multifactorial Adaptive Platform Trial for Community-Acquired Pneumonia) platform of 2 multicenter trials [20, 23]. The remaining studies reported rates of symptomatic VTE (n = 4207) (Table 2). Risk of VTE was consistently reduced with intensified anticoagulation compared with prophylaxis (2.8% [81/2888] vs 5.4% [151/2794]; RR, 0.53 [95% CI, .41–.69]; *I*^2^ = 0%, 8 studies) (Figure 2*B*). The effect was driven by a reduction in PE (1.3% [37/2801] vs 3.5% [95/2708]; RR, 0.39 [95% CI, .27–.57]; *I*^2^ = 0%) but not DVT (1.3% [36/2801] vs 1.7% [47/2708]; RR, 0.81 [95% CI, .48–1.35]; *I*^2^ = 21%) (Supplementary Figures 4 and 5). Intensified anticoagulation was also associated with a reduction in the composite outcome of thrombosis or death (4 studies; RR, 0.78 [95% CI, .66–.91]; *I*^2^ = 0%) (Supplementary Figure 6). Risk for any thrombosis was reduced (Supplementary Figure 7), but without evidence of effect on arterial thrombosis (8 studies; RR, 1.26 [95% CI, .57–2.77]; *I*^2^ = 50%).

### Safety Outcomes

Risk of major bleeding was increased with intensified anticoagulation compared with prophylaxis (2.3% [69/3004] vs 1.3% [38/2869]; RR, 1.73 [95% CI, 1.17–2.56]; *I*^2^ = 0%; 11 studies) (Figure 2*C*). Risk of clinically relevant nonmajor bleeding (4.4% vs 1.9%; 7 studies; RR, 2.08 [95% CI, 1.13–3.83]; *I*^2^ = 11%) and any bleeding (8.8% vs 4.3%; 7 studies; RR, 1.90 [95% CI, 1.16–3.12]; *I*^2^ = 30%) was also increased with use of intensified anticoagulation (Supplementary Figures 7–11).

### Subgroup Analysis

**Figure 3.**
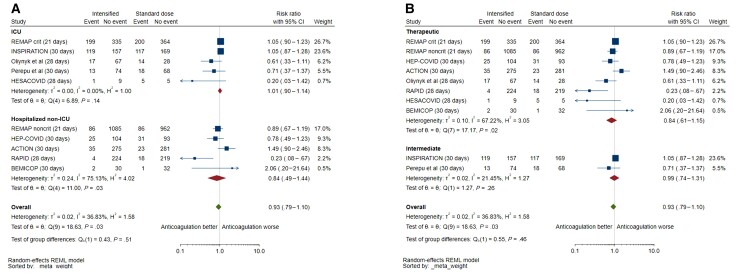
Subgroup analysis of mortality with intensified vs prophylactic anticoagulation, by clinical setting (intensive care unit [ICU] vs hospitalized non-ICU) (*A*) and by dose of intensified anticoagulation (therapeutic vs intermediate) (*B*). The single outpatient trial [27] was excluded from the forest plot because of no mortality events. Two other trials were excluded because venous thromboembolic events were not captured as outcomes [30, 31]. Abbreviations: CI, confidence interval; ICU, intensive care unit; REML, restricted maximum likelihood.

There was a signal of mortality reduction for inpatients admitted to general wards, although with low precision and high heterogeneity (5 studies; RR, 0.84 [95% CI, .49–1.44]; *I*^2^ = 75%); this effect was not significantly different to studies performed in the ICU (interaction *P* = .51) (Figure 3*A*). There was also no difference in effect between therapeutic and intermediate dosing on mortality (interaction *P* = .46), but substantial heterogeneity existed between trials testing therapeutic doses (*I*^2^ = 67%, *P* = .02) (Figure 3*B*). There was insufficient subgroup data to analyze the effect of intensified unfractionated heparin on mortality. Exclusion of trials using predominantly nonheparin anticoagulants (ACTION [AntiCoagulaTloncOroNavirus trial] and ACTIV-4b (Accelerating COVID-19 Therapeutic Interventions and Vaccines, Outpatient Thrombosis Prevention trial) showed no effect on mortality (9 studies; RR, 0.88 [95% CI, .73–1.06]).

**Figure 4.**
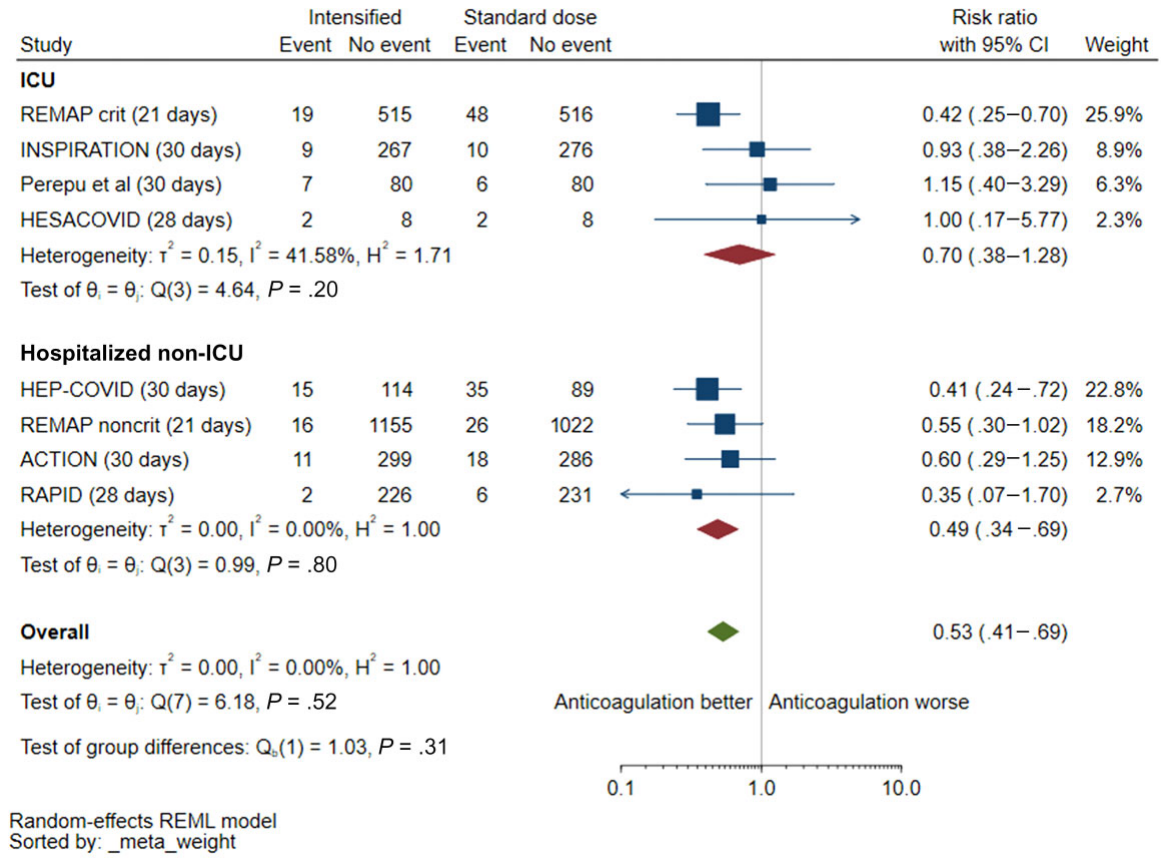
Venous thrombosis with intensified vs prophylactic anticoagulation, by stratified by clinical setting (intensive care unit [ICU] vs hospitalized non-ICU). The single outpatient trial [27] was excluded from the forest plot because of no mortality events. Two other trials were excluded because venous thromboembolic events were not captured as outcomes [30, 31]. Abbreviations: CI, confidence interval; ICU, intensive care unit; REML, restricted maximum likelihood.

Pooled VTE risk reduction was greater in studies conducted in hospitalized non-ICU settings (4 studies; RR, 0.49 [95% CI, .34–.69]) compared with those done in ICU (4 studies; RR, 0.70 [95% CI, .38–1.28]), but this difference was not statistically significant (interaction *P* = .31) (Figure 4). This effect was seen in trials using therapeutic anticoagulation (6 studies; RR, 0.47 [95% CI, .36–.63]) but not those testing intermediate-dose anticoagulation (2 studies; RR, 1.02 [95% CI, .52–2.0]; interaction *P* = .04) (Supplementary Figure 12). In an exploratory analysis, there was no reduction in mortality with intensified anticoagulation in both trials showing a significant reduction in VTE events among non–critically ill patients [23, 26] (n = 2472; RR, 0.86 [95% CI, .67–1.10]; *I*^2^ = 0%) or in trials without a clear VTE effect (RR, 0.62 [95% CI, .10–3.87]; *I*^2^ = 90%).

**Figure 5.**
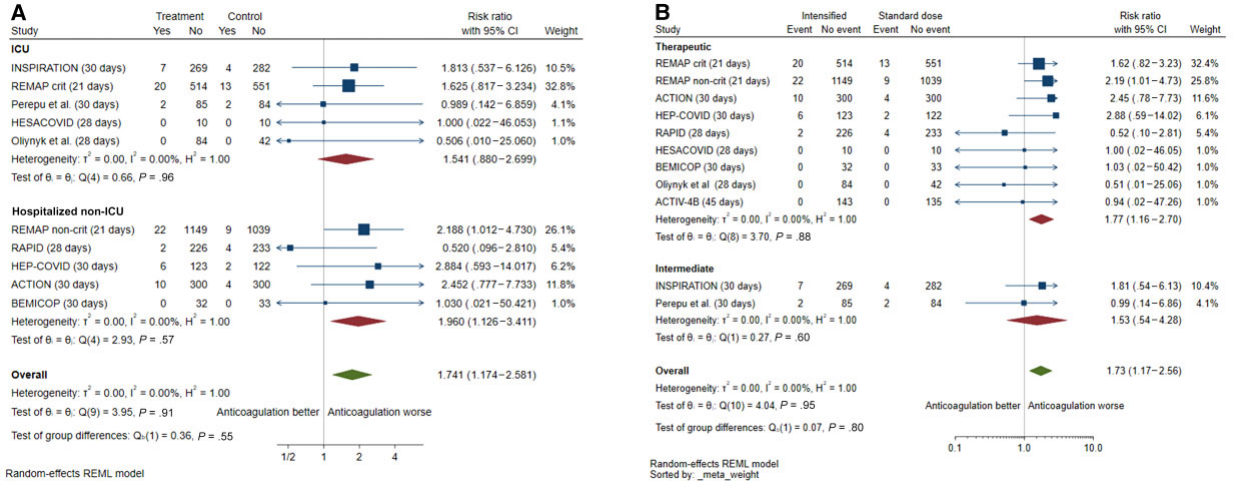
Subgroup analysis of major bleeding with intensified vs prophylactic anticoagulation, by clinical setting (intensive care unit [ICU] vs hospitalized non-ICU) (*A*) and by dose of intensified anticoagulation (therapeutic vs intermediate) (*B*). Abbreviations: CI, confidence interval; ICU, intensive care unit; REML, restricted maximum likelihood.

Similar increases in major bleeding were observed among critically ill and non–critically ill patients (interaction *P* = .55) and those receiving therapeutic vs intermediate anticoagulant dosing (interaction *P* = .80) (Figure 5*A* and 5*B*).

## DISCUSSION

The data from this meta-analysis, synthesizing outcomes from 11 RCTs involving 5873 adults, show that intensified anticoagulation did not reduce short-term mortality (up to 45 day) for hospitalized patients with COVID-19. This finding was consistent across the spectrum of clinical severity and anticoagulant dosing strategies. Intensified anticoagulation reduced VTE as well as the composite outcome of VTE and death, but at a cost of significantly increased risk of major bleeding.

COVID-19 pneumonia is associated with a hypercoagulable state resulting from endothelial perturbation and an intense prothrombotic inflammatory response [32]. This may progress to a distinct syndrome, termed COVID-19–associated coagulopathy, characterized by markedly elevated D-dimer and fibrinogen concentrations and pulmonary microvascular thrombosis, which has been linked with worse outcome [5, 7–11, 33, 34]. VTE is common even with use of standard-dose thromboprophylaxis, possibly occurring at higher rates than other respiratory conditions [1]. Given the prominence of thrombo-inflammation in the pathogenesis of COVID-19 and the likelihood that pulmonary thrombotic complications contribute to progressive hypoxic respiratory failure, one might expect that by preventing VTE, intensified dosing of anticoagulation should reduce disease severity and related mortality. The lack of overall survival benefit despite significant reduction in VTE events with intensified anticoagulation observed across high-quality trials in our meta-analysis therefore requires explanation.

Our findings are consistent with evidence from medical inpatients without COVID-19, where thromboprophylaxis has established benefit for preventing VTE regardless of risk and illness severity [35–37], but does not reduce mortality and its effect on other important clinical outcomes, such as symptomatic PE, is uncertain [38]. Several factors could play a role in this apparent paradox. Most trials of anticoagulation, including for COVID-19, are not powered to detect a difference in mortality, and absence of an effect on this outcome may result from type 2 error rather than true lack of efficacy. Related to this, thrombotic events, often ascertained as venographic DVT with uncertain clinical significance, are inadequate as a surrogate for efficacy outcomes in thromboprophylaxis trials because of poor correlation with important outcomes [39]—although prophylaxis prevents thrombotic events overall, trials may fail to detect an effect on fatal PE.

There are plausible biological explanations for true absence of mortality effect. The increased risk of major bleeding associated with thromboprophylaxis—80% for standard heparin doses in the most recent Cochrane review [38] and an additional 74% increased risk from intensified anticoagulation for COVID-19 in our analysis—may offset any reduction in mortality due to VTE. Although risk of overt bleeding from intensified anticoagulation was increased in both non-ICU and ICU settings, alveolar hemorrhage, which has been documented in COVID-19–associated acute respiratory distress syndrome (ARDS) [40], may also contribute to overall harm, especially in the latter group. Another possibility is that intensified prophylaxis, even at therapeutic doses, may not lead to reduction in fatal PE and translate into mortality benefit. This is especially relevant in ICU settings where a larger proportion of non-VTE-attributable deaths occur and the presence of ARDS-associated pulmonary microvascular thrombosis (“immunothrombosis”) may be refractory to heparin therapy. Although intensified anticoagulation does reduce PE events this may not an important cause of death in COVID-19, limiting impact on mortality.

An advantage of meta-analysis is the potential to identify subgroups not observed in individual trials that may benefit from an intervention. Our analysis found significant reductions in VTE only in trials that included non–critically ill patients (which all provided therapeutic doses of anticoagulation); this was accompanied by a signal of mortality reduction not seen in trials conducted in the ICU, although with significant between-study heterogeneity. Smaller meta-analyses investigating anticoagulation in COVID-19 have also reported a trend toward reduced mortality in non–critically ill patients only [41–43]. These findings suggest that a window may exist earlier in the disease course of COVID-19 for optimal timing of anticoagulation to prevent VTE and avert disease progression via reduction of pulmonary microthrombosis and pleotropic effects of heparin. The average number of days from symptom onset to hospitalization or enrollment ranged from 1.4 to 10 days among included studies in our review, and 4 of the 5 trials in non-ICU settings required elevated D-dimer or other indicator of coagulopathy for enrollment. These patients may have already developed COVID-19–associated coagulopathy, possibly missing a crucial intervention period where benefit of anticoagulation may be maximized. Currently, however, the absence of demonstrable effect on mortality coupled with significantly increased bleeding risk (which includes intracranial and fatal bleeding in some trials) does not justify introduction of intensified anticoagulation into routine care for non–critically ill patients with COVID-19 pneumonia.

Existing data also do not provide clear guidance for an optimal anticoagulation dosing strategy that balances risk of bleeding with clinical benefit. On subgroup analysis, the largest effect on VTE reduction (Supplementary Figure 12) was seen with therapeutic doses of anticoagulation. Bleeding risk was statistically similar across dosing groups, but the precision was low for intermediate dosing and the established dose-response relationship for bleeding with heparin raises concerns about use of therapeutic dosing. There are currently no RCT data on use of intermediate-dose anticoagulation for COVID-19 in non–critically ill adults, who appeared to derive the most benefit from anticoagulation. Although VTE reduction was only apparent in trials using therapeutic anticoagulation, observational studies have suggested mortality benefit and lower bleeding risk from intermediate-dose anticoagulation among hospitalized COVID-19 patients, with a high representation of patients from general wards [44, 45]. Ongoing trials predominantly enrolling non–critically ill adults will inform the role and optimal use of intensified prophylaxis in COVID-19: ASCOT (NCT04483960, n = 2400, therapeutic and intermediate LMWH vs standard prophylaxis); PROTHROMCOVID (NCT04730856, n = 600, therapeutic and intermediate tinzaparin vs standard prophylaxis); INHIXACOV19 (NCT04427098, n = 300, intermediate vs prophylactic dose enoxaparin); XACT (NCT04640181, n = 150, therapeutic or intermediate enoxaparin or rivaroxaban vs standard prophylaxis); ACT (NCT04324463, n = 6000, aspirin and rivaroxaban vs standard of care); and FREEDOM COVID (NCT04512079, n = 3600, therapeutic enoxaparin vs enhanced-dose rivaroxaban vs prophylaxis).

This review has several limitations. First, we analyzed trial-level data, limiting the extent to which we could explore differences in subgroups by important baseline prognostic variables such as age, comorbidity, and markers of disease severity and inflammation. Second, although we performed subgroup analysis by clinical setting (as a surrogate for disease severity), criteria for severe disease and ICU eligibility were institution- and study-specific, limiting generalizability. This may have contributed to the extreme heterogeneity (*I*^2^ = 75%) observed among non-ICU-based studies in the risk ratios for mortality. Third, the relatively small number of events limited precision of effect estimates, especially for the non–critically ill subgroup where there was possibly a signal for reduced mortality. We were not able to analyze effect of intensified anticoagulation on need for, and duration of, organ support since these outcomes were not consistently reported. Fourth, we identified 2 studies to be at high risk of bias and with some concerns, chiefly with regard to trials using nonobjective methods in defining and detecting thrombosis events. This serves to emphasize the limitation using of thrombotic events as an outcome in anticoagulation trials. Fifth, asymmetry in the funnel plots indicates possibility of publication bias, but the small number of included trials limits accuracy. Finally, although sensitivity analysis showed no effect modification on the primary outcome with omission of individual trials, this meta-analysis was dominated by events from 2 large multicenter studies [20, 23] in which a large proportion of patients in the usual-care groups received intermediate-dose prophylaxis. This may have skewed the effect of intensified anticoagulation toward the null; 1 recent systematic review showed a more precise effect of anticoagulation on mortality (albeit still nonsignificant) among moderately ill patients after excluding these trials [25].

In conclusion, available data indicate that intensified anticoagulation has no effect on short-term mortality among hospitalized adults with COVID-19 and is associated with increased risk of bleeding. The finding of significant reductions in VTE with a possible signal for reduced mortality in non-ICU hospitalized adults suggests that additional studies, with a focus on moderately ill patients and different dosing strategies, may delineate optimal use of thromboprophylaxis in this condition.

## Supplementary Data

Supplementary materials are available at *Open Forum Infectious Diseases* online. Consisting of data provided by the authors to benefit the reader, the posted materials are not copyedited and are the sole responsibility of the authors, so questions or comments should be addressed to the corresponding author.

## Notes

***Author contributions.*** Conception and writing of protocol: N. K. W., S. W., J. E. Registration of protocol on PROSPERO: N. N., K. P., O. S. Record screening, data extraction, and risk of bias assessment: N. K. W., N. N., K. P., O. S., and M. A. Analysis and interpretation and drafting of the manuscript: N. K. W., S. W., J. E. Critical review of the manuscript: All authors.

***Acknowledgments.*** The authors thank the University of Cape Town Health Sciences Library for assistance with development of search terms and strategy.

***Financial support*.** This work was supported by the Wellcome Trust through core funding from the Wellcome Centre for Infectious Diseases Research in Africa (203135/Z/16/Z). S. W. was supported by the National Institutes of Health (K43TW011421). For the purpose of Open Access, the author has applied a CC BY public copyright license to any Author Accepted Manuscript version arising from this submission.

***Potential conflicts of interest*.** The authors: No reported conflicts of interest.

All authors have submitted the ICMJE Form for Disclosure of Potential Conflicts of Interest. Conflicts that the editors consider relevant to the content of the manuscript have been disclosed.

## Supplementary Material

ofac285_Supplementary_Data
