# Supplementary material for: Efficacy and Safety of Intensified Versus Standard Prophylactic Anticoagulation Therapy in Patients With Coronavirus Disease 2019: A Systematic Review and Meta-Analysis
Source: Open Forum Infect Dis. 2022 Jun 7;9(7):ofac285. doi: 10.1093/ofid/ofac285 (PMC9214161; doi:10.1093/ofid/ofac285)
Supplement: ofac285_Supplementary_Data [file ofac285_supplementary_data.zip › Supplementary figures_25May2022_OFID.docx]

**
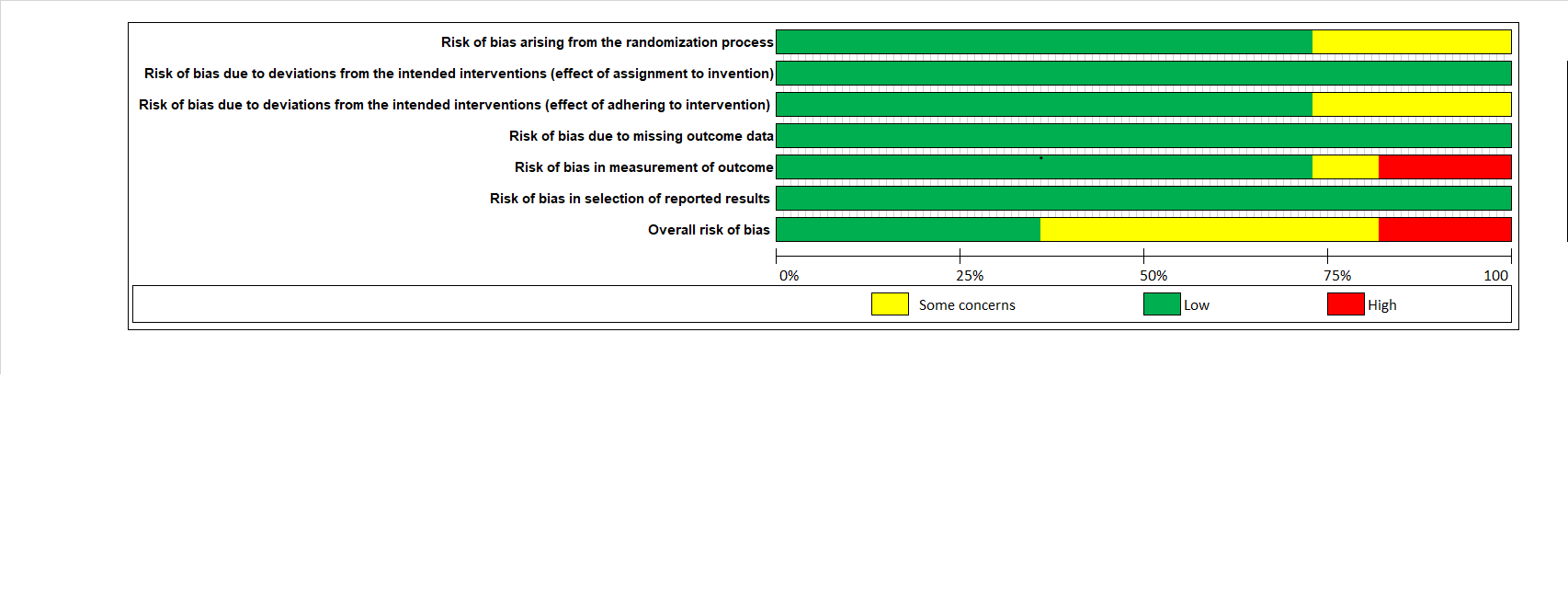
**

**Supplementary Figure S1. Risk of bias graph: review authors' judgements about each risk of bias item presented as percentages across all included studies.**

**
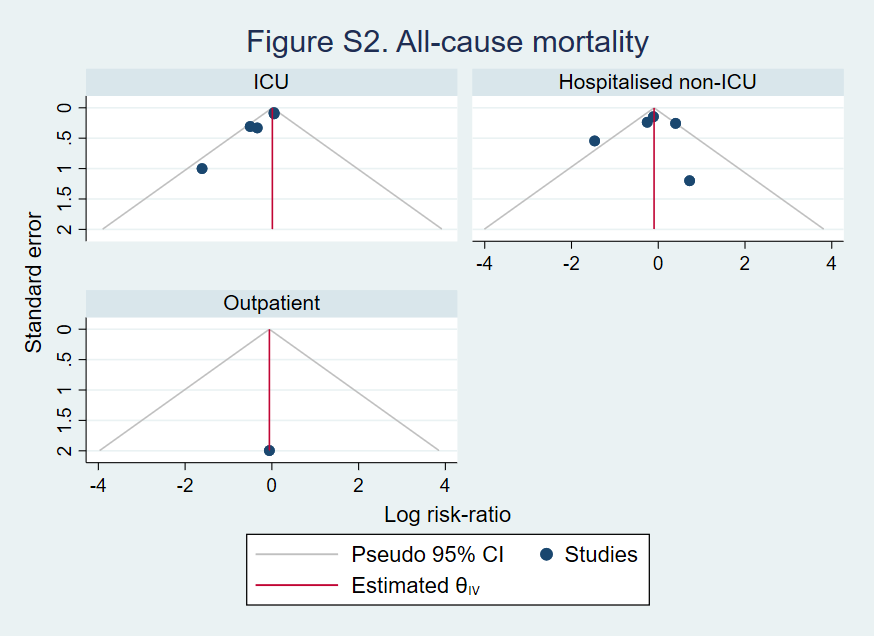
**

**Supplementary Figure S2. Funnel plot for mortality in groups receiving intensified versus prophylactic anticoagulation, stratified by clinical setting (ICU, hospitalised non-ICU and outpatient) (n = 11 studies)**


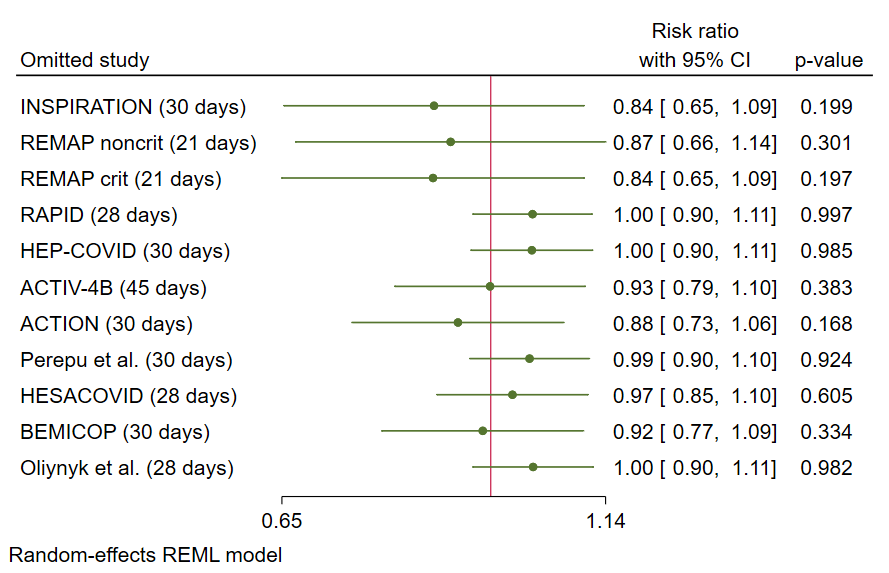


**Supplementary Figure S3. Leave-one-out forest plot, demonstrating overall effect size computed from meta-analysis with each individual trial excluded (n = 11 studies)**

**
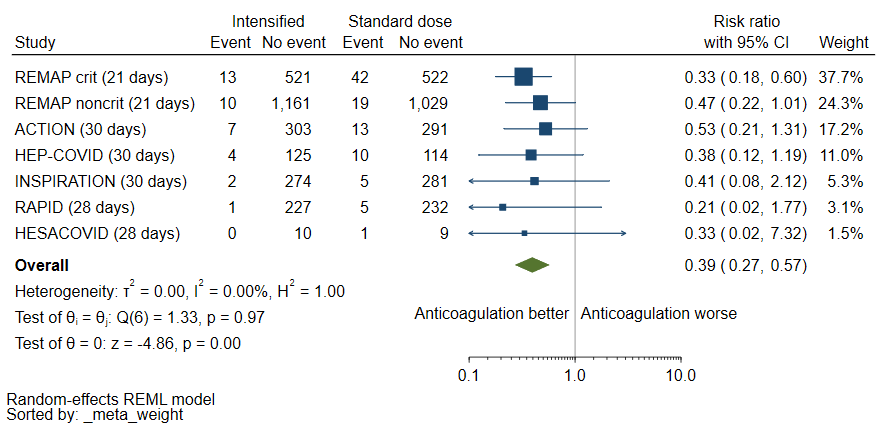
**

**Supplementary Figure S4. Forest plot of risk ratio for pulmonary embolism (PE) in groups receiving intensified versus prophylactic anticoagulation**

**(n = 7 studies).** There were no thrombotic events in the single outpatient trial ^1^ and the pulmonary embolic events were not captured as outcomes in the remaining trials ^2–4^ – these trials are excluded from the forest plot above.


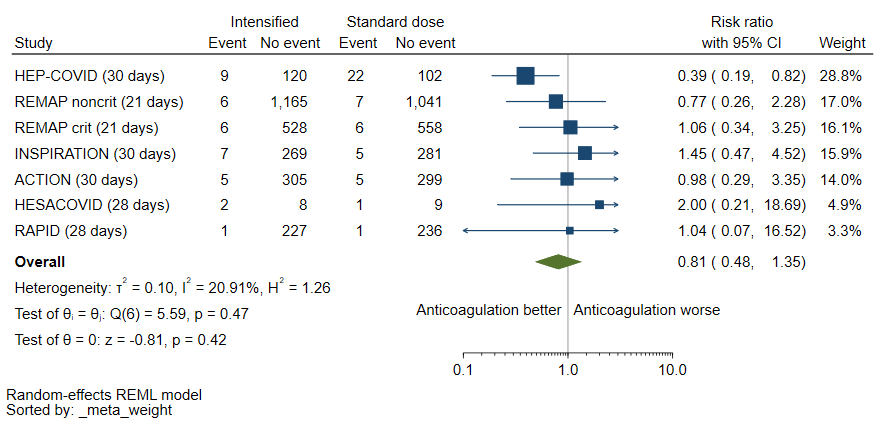


**Supplementary Figure S5. Forest plot of risk ratio for deep venous thrombosis in groups receiving intensified versus prophylactic anticoagulation**

**(n = 7 studies).** There were no thrombotic events in the single outpatient trial ^1^ and DVT events were not captured as outcomes in the remaining trials ^2–4^ – these trials are excluded from the forest plot above.

**
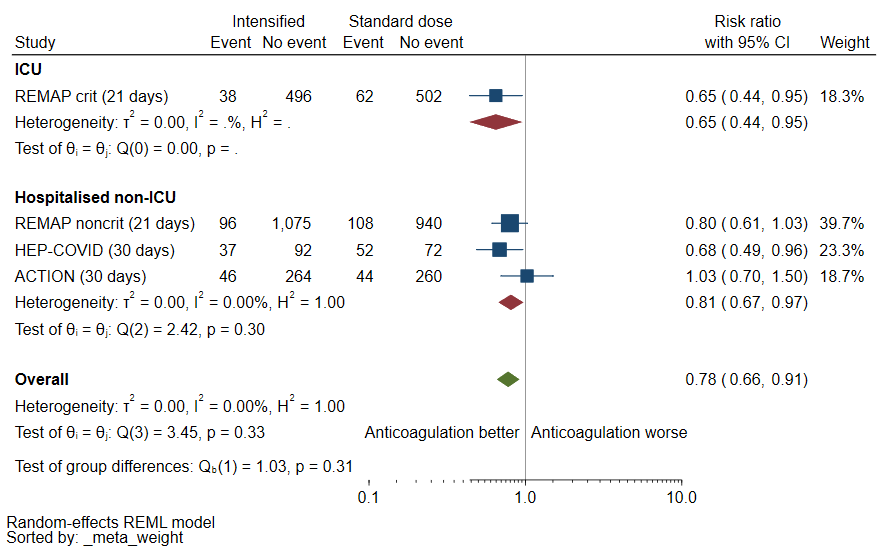
**

**Supplementary Figure S6. Forest plot of risk ratio for composite outcome (any thrombosis or death) in groups receiving intensified versus prophylactic anticoagulation, stratified by clinical setting (ICU versus hospitalised non-ICU) (n = 4 studies).** Only 4 studies reported on this composite outcome, ^5–8^ all comparing therapeutic anticoagulation to low or intermediate-dose prophylactic anticoagulation.


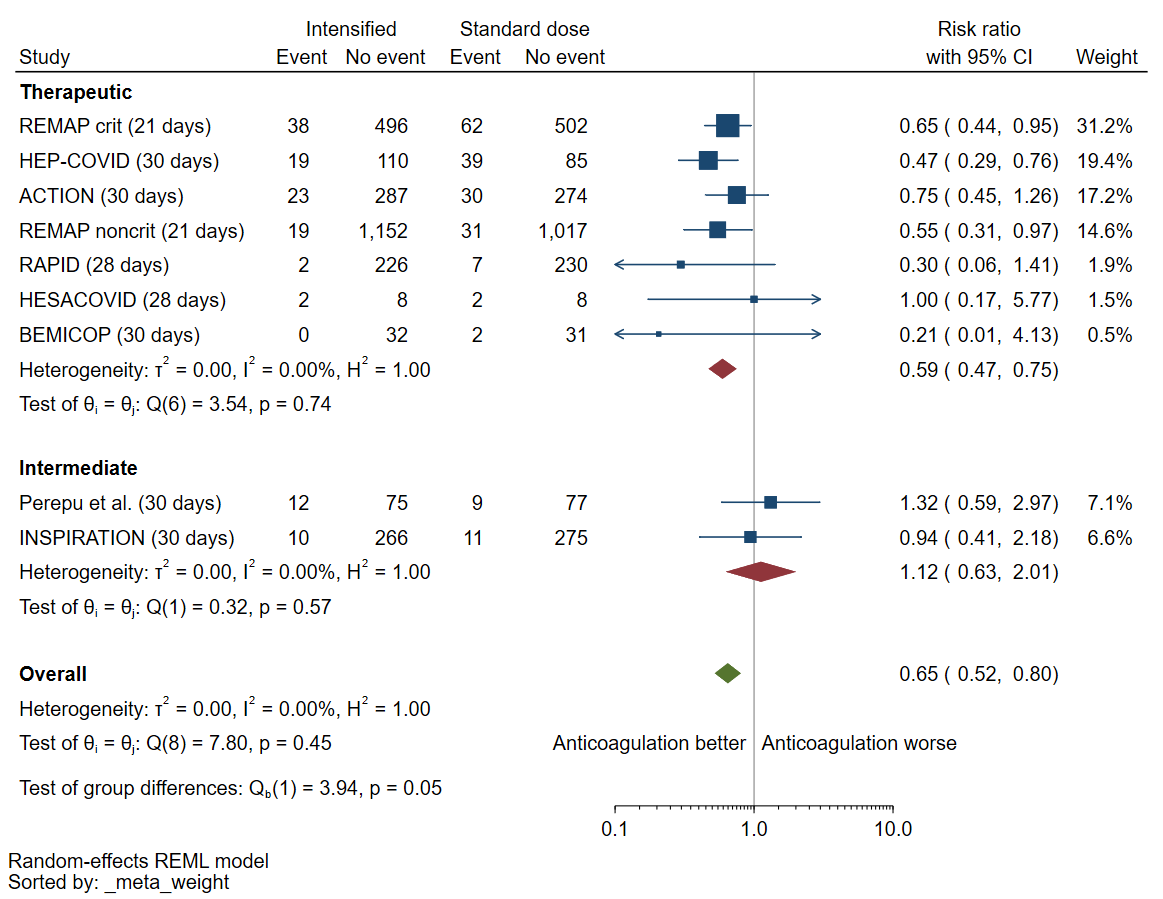


**Supplementary Figure S7. Forest plot of risk ratio for any thrombosis in groups receiving intensified versus prophylactic anticoagulation, stratified by dose of intensified anticoagulation (therapeutic versus intermediate) (n = 9 studies).** There were no thrombotic events in the single outpatient trial ^1^ and thrombotic events were not captured as an outcome in another trial ^3^ – these trials are excluded from the forest plot above.

**
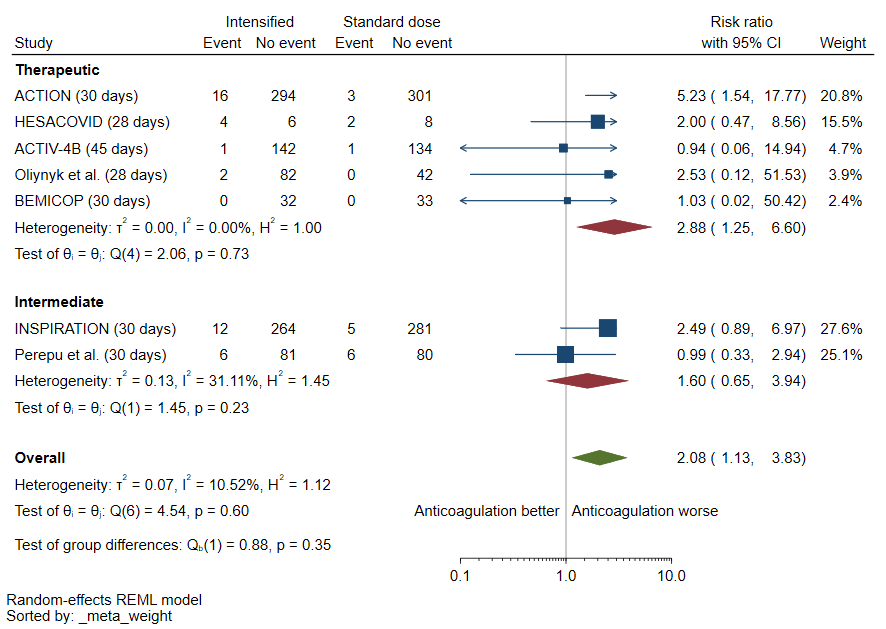
**

**Supplementary Figure S8. Forest plot of risk ratio for clinically relevant non-major bleed in groups receiving intensified versus prophylactic anticoagulation, stratified by dose of intensified anticoagulation (therapeutic versus intermediate) (n = 7 studies).** This outcome was not reported by 4 trials ^5,6,8,9^ excluded from forest plot above.

**
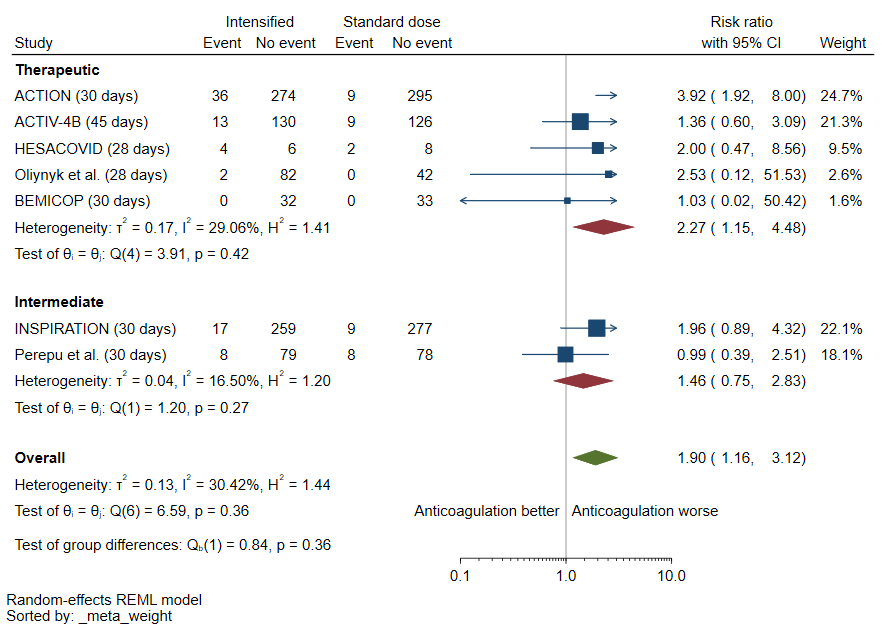
**

**Supplementary Figure S9. Forest plot of risk ratio for any bleed in groups receiving intensified versus prophylactic anticoagulation, stratified by dose of intensified anticoagulation (therapeutic versus intermediate) (n = 7 studies).** This outcome was not reported by 4 trials ^5,6,8,9^ excluded from forest plot above.

**
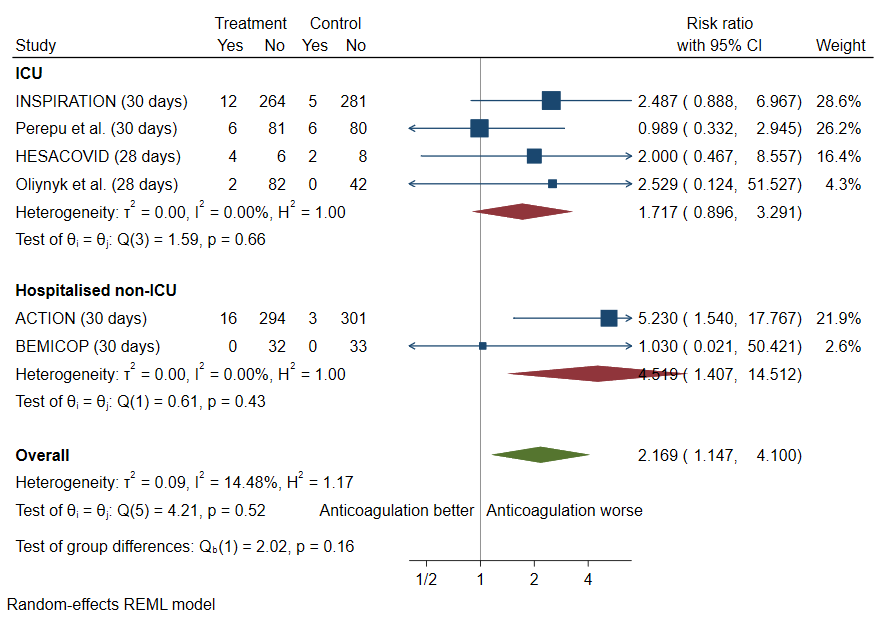
**

**Supplementary Figure S10. Forest plot of risk ratio for clinically relevant non-major bleed in groups receiving intensified versus prophylactic anticoagulation, stratified by clinical setting (ICU versus hospitalised non-ICU) (n = 6 studies).** This outcome was not reported by 4 in-hospital trials ^5,6,8,9^ excluded from the forest plot above.


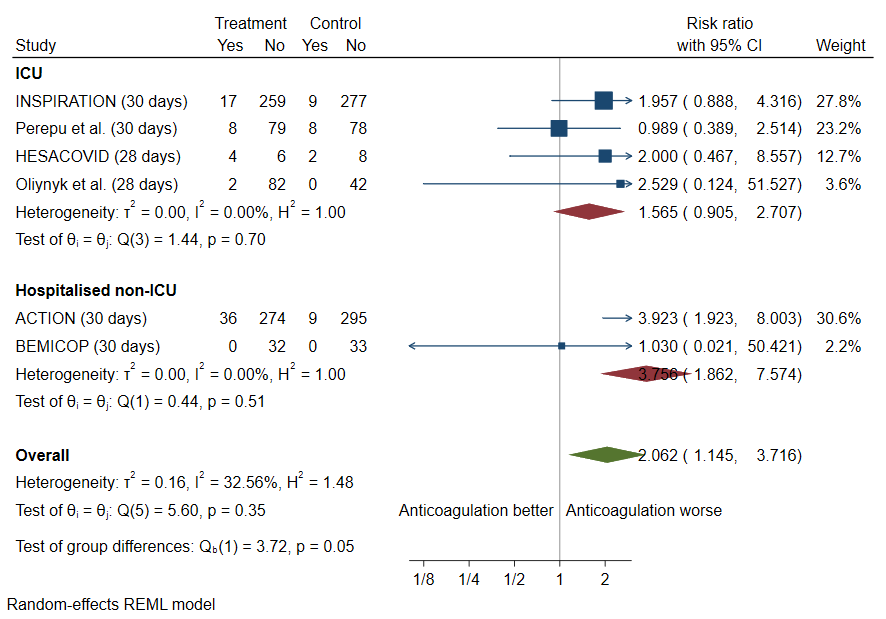


**Supplementary Figure S11. Forest plot of risk ratio for any bleed in groups receiving intensified versus prophylactic anticoagulation, stratified by clinical setting (ICU versus hospitalised non-ICU)(n = 6 studies).** This outcome was not reported by 4 in-hospital trials ^5,6,8,9^ excluded from the forest plot above.

**
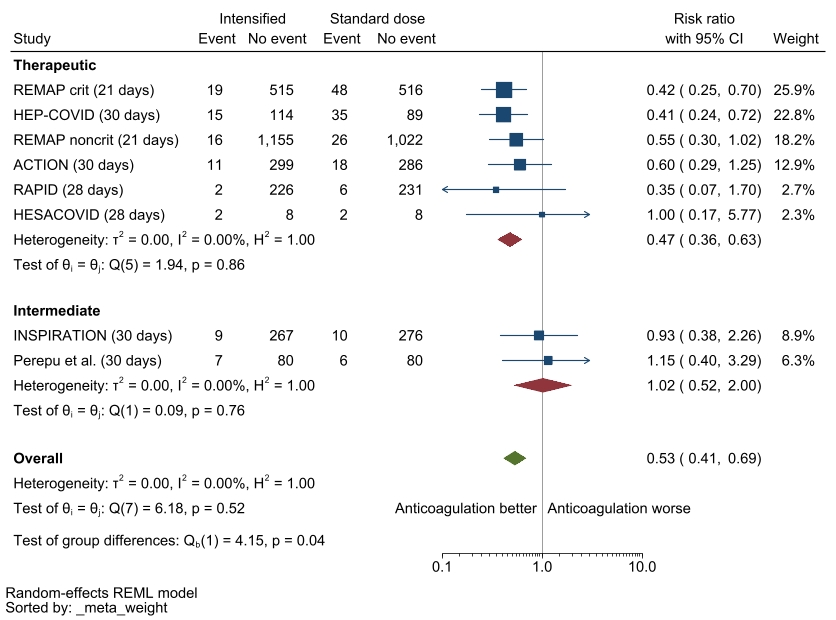
**

**Supplementary Figure S12. Forest plot of risk ratio for venous thrombosis in groups receiving intensified versus prophylactic anticoagulation, stratified by dose of intensified anticoagulation (therapeutic versus intermediate) (n = 8 studies).** There were no reported thrombotic events in the single outpatient trial ^1^ and venous thromboembolic events were captured as outcomes in two trials ^2,3^ – these trials are excluded from the forest plot above.

# **References**

1. Connors JM, Brooks MM, Sciurba FC, et al. Effect of Antithrombotic Therapy on Clinical Outcomes in Outpatients with Clinically Stable Symptomatic COVID-19: The ACTIV-4B Randomized Clinical Trial. *JAMA - J Am Med Assoc*. 2021;326(17):1703-1712. doi:10.1001/jama.2021.17272

2. Marcos M, Carmona-Torre F, Vidal Laso R, et al. Therapeutic vs. prophylactic bemiparin in hospitalized patients with non-severe COVID-19 (BEMICOP): an open-label, multicenter, randomized trial. *Thromb haemost*. Published online 2021. doi:10.1055/a-1667-7534

3. Oliynyk O, Barg W, Slifirczyk A, et al. Comparison of the effect of unfractionated heparin and enoxaparin sodium at different doses on the course of covid-19-associated coagulopathy. *Life*. 2021;11(1032). doi:10.3390/life11101032

4. Perepu US, Chambers I, Wahab A, et al. Standard prophylactic versus intermediate dose enoxaparin in adults with severe COVID-19: A multi-center, open-label, randomized controlled trial. *J Thromb Haemost*. 2021;19(9):2225-2234. doi:10.1111/jth.15450

5. The REMAP-CAP, ACTIV-4a and AI. Therapeutic Anticoagulation with Heparin in Critically Ill Patients with Covid-19. *N Engl J Med*. Published online 2021:1-13. doi:10.1056/nejmoa2103417

6. The REMAP-CAP, ACTIV-4a and AI. Therapeutic Anticoagulation with Heparin in Noncritically Ill Patients with Covid-19. *N Engl J Med*. Published online 2021:1-13. doi:10.1056/nejmoa2105911

7. Lopes RD, de Barros E Silva PGM, Furtado RHMM, et al. Therapeutic versus prophylactic anticoagulation for patients admitted to hospital with COVID-19 and elevated D-dimer concentration (ACTION): an open-label, multicentre, randomised, controlled trial. *Lancet*. 2021;397(10291):2253-2263. doi:10.1016/S0140-6736(21)01203-4

8. Spyropoulos AC, Goldin M, Giannis D, et al. Efficacy and Safety of Therapeutic-Dose Heparin vs Standard Prophylactic or Intermediate-Dose Heparins for Thromboprophylaxis in High-risk Hospitalized Patients with COVID-19: The HEP-COVID Randomized Clinical Trial. *JAMA Intern Med*. 2021;181(12):1612-1620. doi:10.1001/jamainternmed.2021.6203

9. Sholzberg M, Tang GH, Rahhal H, et al. Effectiveness of therapeutic heparin versus prophylactic heparin on death, mechanical ventilation, or intensive care unit admission in moderately ill patients with covid-19 admitted to hospital: RAPID randomised clinical trial. *BMJ*. 2021;375. doi:10.1136/bmj.n2400
